# Birt–Hogg–Dubé syndrome: a literature review and case study of a Chinese woman presenting a novel FLCN mutation

**DOI:** 10.1186/s12890-017-0383-9

**Published:** 2017-02-21

**Authors:** Shengyu Hao, Fei Long, Fenglan Sun, Teng Liu, Daowei Li, Shujuan Jiang

**Affiliations:** Department of Respiratory Medicine, Shandong Provincial Hospital affiliated to Shandong University, 324 Jingwu Road, Jinan, Shandong 250021 China

**Keywords:** Birt–Hogg–Dubé syndrome, Mutation, Lung bubblae, Pneumothorax, Treatment

## Abstract

**Background:**

The Birt–Hogg–Dubé (BHD) syndrome is a very rare autosomal dominant form of genodermatosis caused by germline mutations in the folliculin (*FLCN*) gene, which is mapped to the p11.2 region in chromosome 17. BHD commonly presents cutaneous fibrofolliculomas, pulmonary cysts, renal cell carcinoma, and recurrent pneumothoraxes. The disease is easily ignored or misdiagnosed as pneumothorax, pulmonary lymphangiomyomatosis (LAM), or emphysema. Follow-up and guidelines for managing recurrent pneumothoraxes in these patients are lacking.

**Case presentation:**

We reported the case of a 56-year-old Chinese woman who presented skin lesions, multiple lung bubblae, recurrent pneumothoraxes, thyroid nodules, and pulmonary inflammatory pseudotumors (PITs). The patient had a family history of pneumothoraxes and renal tumor. The BHD diagnosis was confirmed by genetic testing, which revealed a novel *FLCN* mutation in exon 14. Furthermore, the patient underwent a bullectomy because of recurrent pneumothorax 6 years ago.

**Conclusion:**

To our knowledge, the novel mutation in exon 14 and the manifestation of PIT in the present case have never been reported for BHD. The patient underwent a bullectomy previously with no relapse at the last follow-up before the preparation of this report, thereby suggesting that thoracotomy with bullectomy may be a possible therapeutic approach for some BHD patients with recurrent pneumothorax.

## Background

BHD syndrome is a rare autosomal dominant form of genodermatosis; the disease characterized by cutaneous fibrofolliculomas, pulmonary cysts, renal cell carcinoma, and recurrent pneumothoraxes [1]. Several other manifestations of BHD have been reported, such as colorectal carcinoma and colon polyps [2–4], thyroid nodules, parotid oncocytomas, multiple lipomas, oral papules, and cancer [5, 6]. However, their causal associations with BHD have not been clinically validated.

BHD was first reported as an inherited dermatological syndrome in 1977; the gene responsible for the syndrome was cloned in 2002 [7, 8]. To date, 152 unique pathogenic FLCN gene mutations in 616 families have been reported in worldwide; approximately 90% of these mutations were reported in Europe and the United States [6]. The most frequent hot spot of mutation occurs in a tract of eight cytosines in exon 11 identified in approximately 50% of BHD patients regardless of ethnicity. Mutations in exons 9 and 12 are common, and their association with lung cysts and pneuomothoraces is statistically significant [1, 5, 9]. Multiple bilateral pulmonary cysts occur in 70 to 84% of the affected members in BHD families [5, 10, 11]. Approximately 30% of BHD patients will develop spontaneous pneumothoraxes, most frequently before the age of 40 years (median age of onset, 38 years) in Europe and the United States [10]. By contrast, multiple pulmonary cysts with recurrent pneumothorax are observed in approximately 90% of the BHD cases in Japanese patients [12]. Although the number of case reports describing lung disorders has increased, data are still lacking on the effective measures and follow-up to support the treatment of pneumothorax in BHD.

We reported the case of a Chinese woman with skin lesions, multiple lung bullae, recurrent pneumothorax, thyroid nodes, PIT, and family history of spontaneous pneumothoraxes and renal tumor. The woman was subsequently diagnosed with a novel mutation. Moreover, the patient underwent a pulmonary bullectomy for recurrent pneumothorax 6 years ago.

## Case presentation

**Fig. 1:**
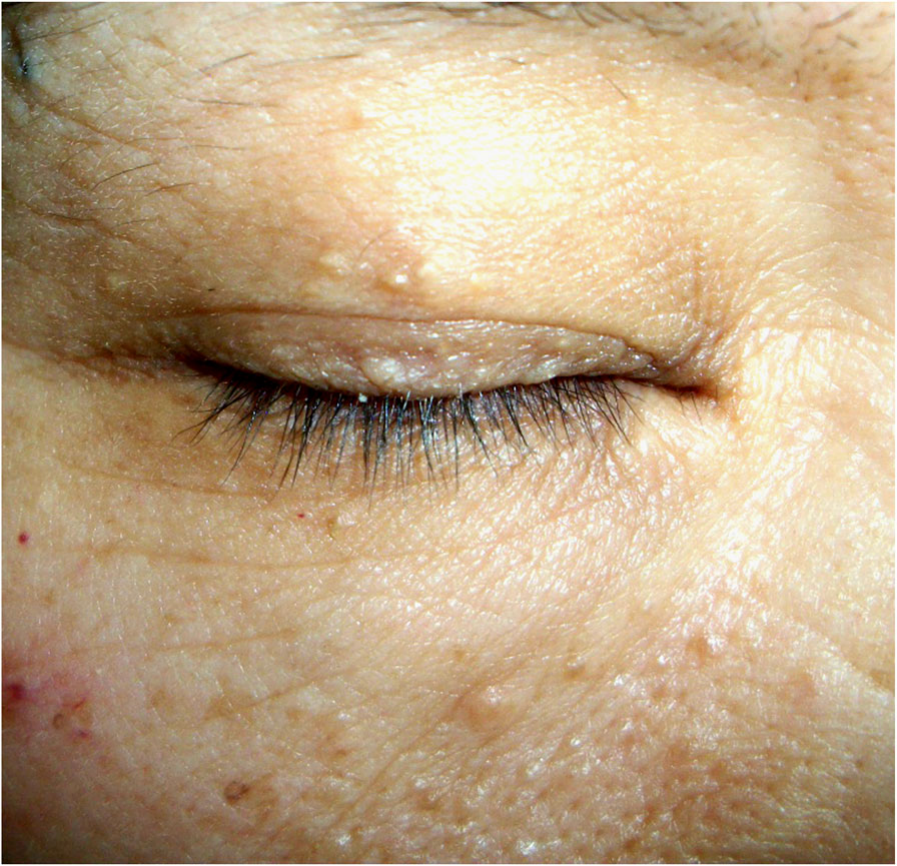
Multiple pale and dome-shaped macules over the patient’s face, which are characteristic skin lesions of BHD syndrome

**Fig. 2:**
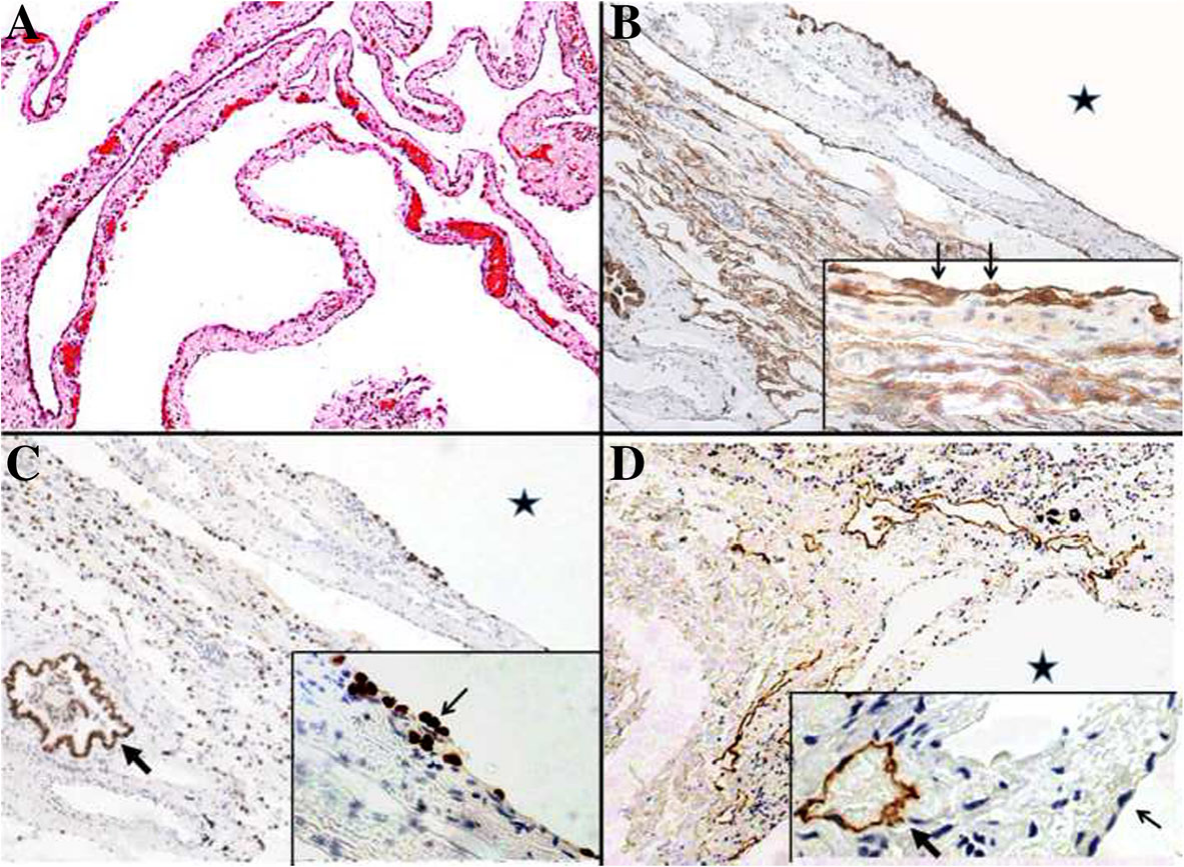
Histopathological features of the resected lung specimens from the patient 6 years ago. Cysts are indicated by *stars*. **a** These cysts are incorporated with interstitial stroma of the interlobular septum or pleura (hematoxylin–eosin–safranin staining). Cysts lined by **b** CK7^+^ and **c** TTF-1^+^ pneumocytes. **d** D2-40 is negative in the lining (*thin arrows*) but positive in lymphatics (*thick arrow*)

A 56-year-old non-smoking Chinese woman visited our hospital; she has cough, expectoration, and throat discomfort for the past 45 days. The patient had no fever, chest congestion, or chest pain. Upon physical examination, multiple white dome-shaped papules were found on her cheek, palpebra, fossa axillaris, and back (Fig. 1). Her medical history showed a tendency to illness. The patient first developed pneumothorax at the age of 36, with multiple bilateral pulmonary cysts and subsequently underwent a tube thoracostomy. After 12 years, the pneumothorax recurred. At 50 years of age, the patient had a third pneumothorax; her right lung was compressed by 40%. Finally, the patient consented to surgical intervention for therapeutic purposes. During surgery, thin-walled bullae with sizes from 0.1 cm to 3.0 cm in diameter were observed on the right lung pleura. The bulla over 10 cm in diameter was found in the right horizontal fissure, and a 2.5 cm -diameter intrapulmonary bulla containing liquid was found in the lateral segment of the right middle lobe. Partial pleura adhesions were released by an electrosurgical generator. The 10 cm bulla was completely removed through wedge-shaped excision. The other bubblae on the pleura were clamped, closed and removal. The incisions were sutured, and the thoracic wall was rubbed and congested for pleurodesis. Histopathological examination showed that the inner surfaces of the cysts were lined with epithelial cells positive for CK7 and TTF-1; the cysts occasionally contained internal septa consisting of alveolar walls (Fig. 2).

**Fig. 3:**
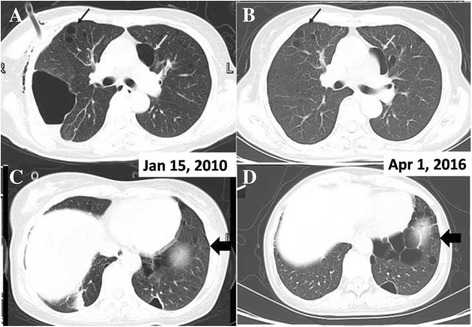
Postoperative changes are shown in the right lung. **a**, **c** The previous CT scan shows numerous cysts in the lung bases; one dominant cyst measures 8.4 cm × 5.1 cm in the right lung and contains some liquid; another huge cyst is anterior to the heart (*white arrow*). **b**, **d** The right lung expanded well after the operation with no obvious scar or atelectasis. Some cysts remained stable (*black arrow*) or became even smaller (*white arrow*). However, more cysts appeared in the lung bases (*thick arrow*)

**Fig. 4:**
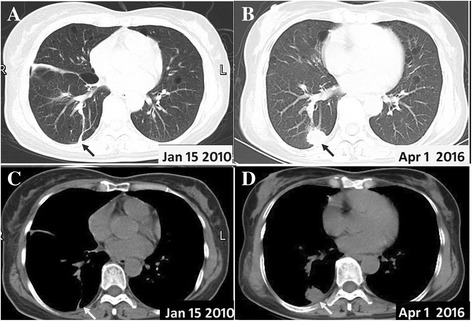
Chest CT scan with pulmonary pseudotumor. **a** Previous CT scan shows a fibrous change in the same slice. **b** The present CT scan shows a 2.8 cm × 2.3 cm pulmonary solid lesion with irregular margins and adjacent pleural thickness. **c**, **d** Corresponding mediastinal window

The patient is a non-smoker, who easily became ill even when she was young. Furthermore, she had a family history of spontaneous pneumothorax. The mother and maternal aunt of the patient had histories of pulmonary bullae and pneumothoraxes; her mother died of renal cancer at the age of 71, with pulmonary bubblae and recurrent pneumothorax. Her 31-year-old daughter and 69-year-old male cousin were classified as BHD-unaffected because they did not show manifestations of BHD upon physical examinations. Her maternal aunt died in her 80s without tumor diseases, but with several recurrent pneumothoraxes. A chest computed tomography (CT) scan taken at our hospital showed multiple cystic lesions throughout the lungs, mainly in the basilar regions (Fig. 3). A 2.8 cm × 2.3 cm solid lesion was present in the posterior basal segement of the lower right lobe (Fig. 4), which was subsequently proven to be a PIT by ultrasound guided percutaneous lung biopsy.

**Fig. 5:**
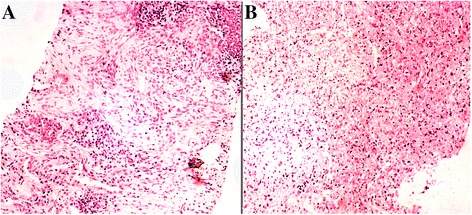
Ultrosound-guided needle biopsy of the lung lesion with immunohistochemistry showed that it is positive for an inflammatory pesudotumor with **a** extensively hyperplasic fibrous and inflammatory cell infiltration, and **b** necrosis lung tissues

Biopsy revealed massive infiltrations of inflammatory cells, including plasma cells and lymphocytes admixed with fibroblasts and myofibroblasts (Fig. 5). Immunohistochemical studies showed positive reactions for CD3, CD20, and Ki-67, whereas no staining was observed with antibodies against CEA, IgG4, IgG, and ALK. These findings were reported as fibrous changes at the same location in the CT performed previously (Fig. 4a, c). Ultrasound examination showed that the patient did not develop a renal carcinoma, but multiple thyroid nodules had emerged.

**Fig. 6:**
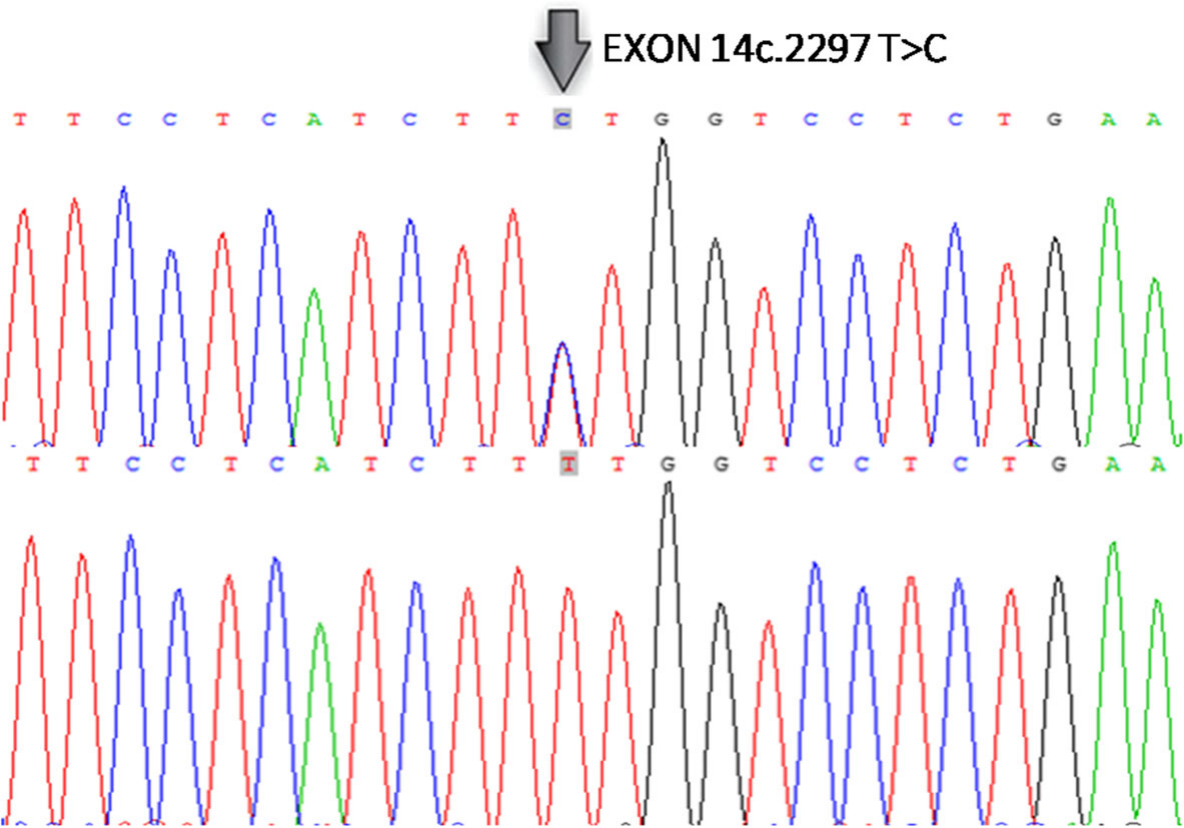
Identification of a germline heterozygous *FLCN* mutation in the patients. The result showed a heterozygous mutation in exon 14 resulting in an animo acid transformation of phenylalanine into serine (p.Phe766Ser)

Given that the patient had multiple skin papules, cystic lesions, pneumothorax, and a family history of pneumothorax and renal tumor, we suspected a congenital disease, particularly BHD. Therefore, we performed blood DNA analysis, and four mutations were identified (Table 1). A mutation of T to C (c.2297 T > C) in exon 14 leads to the change from Phe to Ser (p.Phe766Ser). To our knowledge, this mutation has never been reported before (Fig. 6). Besides the mutation in exon 14, we also detected a mutation in exon 1 (c.-299C > T) with a mutation rate of 0.42. Another mutation in intron 8 (c.871 + 36G > A), with a mutation rate of 0.17, causes the codon 303 mutation (p.G ly303Arg) in *FLCN* 2 transcription. The third mutation in intron 9 (c.1062 + 6C > T) has mutation rate of 0.66. However, these three mutations were previously reported to have a minimal correlation to the onset of BHD [13].

**Table 1:** Summary of total mutations identified in the *FLCN* gene by sequencing

dbSNP	mRNA position^a^	Location or △AA^a^	Alleles^a^	Minor Allele	MAF in CHB
rs1708629	c.-299	5′UTR	C > T	C	0.42
rs3744124	c.871 + 36	Intron 8	G > A	G	0.17
rs8065832	c.1062 + 6	Intron 9	C > T	C	0.66
p.F766S	c.2297	Exon 14	T > C	T	None

△*AA* change in amino acid, *MAF* minor allele frequency, *CHB* samples from Han Chinese in Beijing, China studied in Hapmap project

^a^Referenced to the positive strand and folliculin isoform 1 [GenBank: NM_144997]

## Discussion and conclusion

BHD syndrome is difficult to diagnose at initial evaluation because of its low morbidity and variable manifestations. BHD is not yet well-known in East Asia, particularly in China. BHD is a rare type of genodermatosis and an autosomal dominant disease caused by *FLCN* mutations. To date, 90% of the reported BHD patients are from Europe and the United States; 82 to 92% of the cases in these regions have characteristic skin symptoms. By contrast, the rate of characteristic symptoms is 20 to 29% in Japanese patients, which may account for the relative difficulty to recognize the disease; the clinical manifestations of BHD may have potential racial differences [6, 14, 15]. Recurrent pneumothorax is observed in approximately 90% of Asian patients [12], which is higher by 60% than in Western countries. However, BHD is easily misdiagnosed as cystic lung disease, particularly LAM, Langerhans cell histiocytosis, and lymphocytic interstitial pneumonia.

Imaging studies may facilitate differential diagnosis. Cysts in BHD cases are round or oval, withthin, visible, and uniform walls. The cysts are typically located in the basilar and mediastinal lung regions, which differ from the typical apical location of smoking-related emphysema and bullae in primary spontaneous pneumothoraxes [16]. The number and size of the cysts are variable; cysts less than 1 cm in diameter are most commonly seen [17]. We confirmed these findings in the present patient (Fig. 3). LAM is the most difficult to differentiate from BHD, particularly when associated with cutaneous and renal involvement. Cysts in BHD and LAM are thin walled, but those in LAM are smaller, more circular, and equally distributed [16, 18]. On the other hand, the prognosis of BHD is better. LAM usually progresses to respiratory insufficiency with increasing lung cysts. However, BHD typically does not result in respiratory failure.

Little is known about the development of pulmonary cysts in BHD patients. Some reviews suggest that numerous cysts appear to remain stable or unruptured because the frequency of pneumothorax in elderly BHD cases is not as high as that in middle-aged patients [19]. In the present case, we compared the CT findings. Some bullae of the right lung were removed by bullectomy 5 years ago without any scars or atelectasis in the recent CT. The changes were evident in the lungs. Some bullae remained stable, whereas others were absorbed as they shrank or disappeared. However, bullae at the bottom became larger (Fig. 3). The pathological findings of the cysts in BHD are non-specific [20], which is also true in the present case (Fig. 2). Almost 15 years have passed since the discovery of FLCN, and increasing numbers of BHD cases have been reported. However, follow-up of these affected patients is still needed.

Our case report described a novel mutation in *FLCN*, and a new manifestation of PIT in lung lesions of BHD. Previous studies showed that *FLCN* serves as a tumor suppressor gene. The germline mutations in *FLCN* are related to BHD pathogenesis. However, clear genotype–phenotype associations have not been reported in BHD [4, 21]. PIT is an uncommon benign lesion in the lungs; this lesion is considered to be an inflammatory or reactive lesion rather than a neoplasm. The exact etiology is unknown, and an immune disorder seems to be an acceptable hypothesis. In the present patient, the poor condition of the lungs, with multiple cysts, recurrent pneumothoraxes, and tube thoracostomy, disturbed the pulmonary immunity, thereby making it easily infected. However, these conditions also exist in other BHD patients who did not develop a PIT. The only difference is that the patient in the present case has a novel mutation in *FLCN*. Given that the exact etiology of PIT is unknown and no exact genotype–phenotype associations of BHD have been reported, more data are still needed.

The correlation of the new manifestation in this patient with the new mutation has not been confirmed. The patient also showed multiple thyroid nodules, which were also reported in almost half of the BHD patients. This patient did not develop any renal tumors at the last follow-up. The patient’s mother died of a renal tumor. Renal tumors are present in 27% of the BHD patients [21]. Therefore, we informed the patient of the possibility that she could be affected by a renal tumor and suggested that she undergo abdominal ultrasound scan or CT annually.

The occurrence of multiple bilateral pulmonary cysts and spontaneous pneumothorax is high in BHD patients [5, 10, 11]. The management of BHD from a pulmonary perspective is focused on the treatment of pneumothorax. Conservative measures (observation, aspiration, and tube thoracostomy) are taken to manage the initial pneumothorax, which is usually undiagnosed during the first instance. However, BHD patients still face a high risk of recurrent pneumothorax, thereby making these patients anxious. Video-assisted thoracoscopy and mechanical or chemical pleurodesis are typically reserved for recurrent or non-healing cases [16, 22], which make it difficult for reoperation and thoracic surgeries in later years. A unique method was reported to prevent recurrent pneumothorax without adhesion to the chest wall, as well as total or partial pleural covering; however, success is heavily dependent on local expertise [23, 24]. Although the number of case reports on lung disorders has increased, data collection on effective measures and follow-up are still lacking to support the treatment of pneumothorax in BHD.

We reported the case of a Chinese woman who underwent pulmonary bulla resection 6 years ago and did not have a recurrent pneumothorax at the last follow-up. Adequate management of bullae on the pulmonary surface, thoracic wall congestion, and hemorrhage during surgery favored the adhesion between the visceral and parietal pleura, thereby contributing to the success of the operation.

## Abbreviations

BHD: Birt-Hogg-Dubé
CT: A chest computed tomography
*FLCN*: Folliculin
LAM: Lymphangiomyomatosis
PIT: Pulmonary inflammatory pseudotumor
